# Transmission Electron Microscopy Data on drusen-like deposits in the retinal degeneration sTg-IRBP: HEL mouse model

**DOI:** 10.1016/j.dib.2018.12.007

**Published:** 2018-12-06

**Authors:** Yi-Hsia Liu, Christine Mölzer, Gillian C. Milne, Lucia Kuffová, John V. Forrester

**Affiliations:** a Institute of Medical Sciences, University of Aberdeen, Aberdeen, UK; b Microscopy and Histology Core Facility, University of Aberdeen, Aberdeen, UK; c Department of Ophthalmology, NHS Grampian, Aberdeen, UK; d University of Western Australia, Lions Eye Institute, Perth, Australia

## Abstract

Histology (H&E) and transmission electron microscopy (TEM) data are provided showing age-related changes in the retinal structure of sTg-IRBP:HEL mice. These include substantial photoreceptor loss, atrophy of the retinal pigment epithelium, Bruch׳s membrane disruption and thickening, along with the presence of drusenoid deposits and changes in basal laminar infoldings. These features resemble some of those key characteristics found in the course of human dry (atrophic) age-related macular degeneration (AMD), particularly with regard to drusen. Hence, we believe the sTg-IRBP:HEL mouse model represents a useful and promising archetype for future study of the mechanism of drusen formation in AMD.

**Specifications table**

**Table:** 

Subject area	*Medicine and Dentistry*
More specific subject area	*Ophthalmology*
Type of data	*Transmission electron microscopy (TEM) and histology (H&E) images.*
How data was acquired	*Light microscope: Carl Zeiss Axioskop 40 with ProgRes XT Core 5 colour digital microscope camera.*
	*Transmission electron microscope: JEOL 1400 plus; AMT UltraVUE camera.*
Data format	*Acquired and analysed.*
Experimental factors	*Eyes and retinas collected from B10.BR and sTg-IRBP:HEL mice.*
Experimental features	*TEM shows age-related retinal degeneration in sTg-IRBP:HEL mice.*
Data source location	*University of Aberdeen, Institute of Medical Sciences, Foresterhill, AB25 2ZD, Aberdeen, UK (57.156669, -2.135100).*
Data accessibility	*The data will be available with this article.*
Related research article	*Liu Y-H, Corbett C, Klaska IP, Makinen K, Nickerson JM, Cornall RJ, Kuffova L, Forrester JV. Partial Retinal Photoreceptor Loss in Transgenic Mouse Due to Reduced Levels of Interphotoreceptor Retinol Binding Protein (IRBP, RBP3). Experimental eye research 172, 54–65.*[1]

**Value of the data**

• Despite being a leading cause of blindness in the UK, resulting in a gradual loss of (central) vision, as well as an economic burden, the underlying pathogenesis of especially dry AMD is not understood.
• The atrophic (dry) form of the disease is more prevalent, and there is no treatment available at the present time. The data in this DIB report offer a new model for dry AMD which will allow researching of therapeutic options.
• In contrast to their WT controls, sTg-IRBP:HEL mice exhibit age-related drusenoid deposits, resembling those seen in human dry AMD.
• This animal model is compatible with the clinical cardinal features of human dry AMD and may prove beneficial for future mechanistic AMD research and therapy.

## 1 Data

**Fig. 1:**
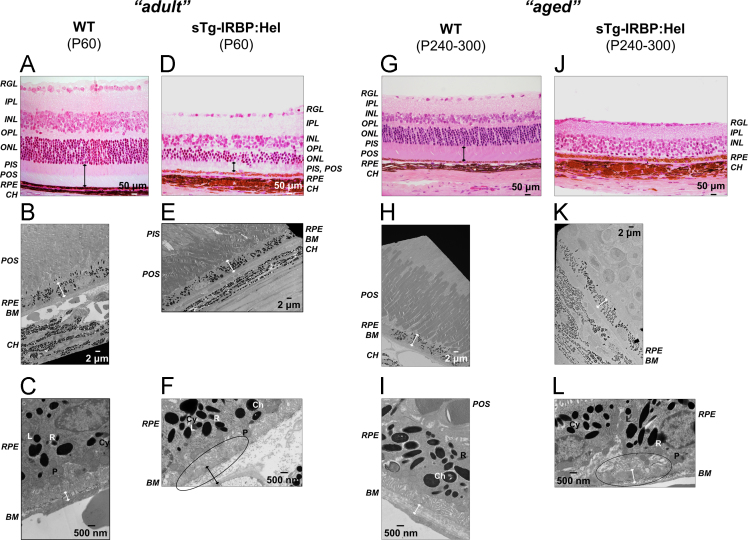
shows representative central retinal sections of 60 day old (P60; “adult”) WT mice (A-C), *vs.* their P60 sTg-IRBP:HEL counterparts (D-F), and of “aged” (P240–300) WT mice (G-I) *vs.* P240–300 sTg-IRBP:HEL mice (J-L). H&E histology and TEM pictures are provided along with medians of *n* = 3 independent measurements per marker. In the adult WT mice, all retinal layers appear complete and intact (A), photoreceptors (180.6 µm) and RPE are well preserved (5.2 µm) (B), and the Bruch׳s membrane shows normal occurrence and thickness (376.3 nm) (C). In the sTg-IRBP:HEL P60 mice, the photoreceptors are partially lost (66.0 µm; D, E), together with BM thickening (1075.0 nm) and abundant drusenoid deposits (F; circle). Comparable to the adult sTg-IRBP:Hel mice, in the aged WT mice (G-I), all retinal layers appear intact, with photoreceptors (118.1 µm) and RPE (4.3 µm) slightly diminished but preserved (G, H). In the aged sTg-IRBP:HEL mice, however, the photoreceptors are completely lost (10.4 µm; J), retinal pigment epithelium further reduced (2.6 µm), and BM thickening and drusenoid deposits are increasingly seen (806.5 nm; K, L; circle). **Circle:** BM membrane disruption with drusenoid deposit and disorganised basal laminar infoldings; **Ch:** charcoal-like granule; **R:** RPE atrophy/loss of pigment; **P:** undigested POS phagosome; **L:** lipofuscin granule; **Cy:** cystic membranous degradation of RPE. Evaluation of retinas for AMD-like features (*i.e.* BM disruption, drusenoid deposits, loss of RPE pigment, lipofuscin deposition, charcoal-like granules, and undigested POS) followed recent reports by Park et al. [3], and Ramkumar et al. [4]. Abbreviations: *RGL*, retinal ganglion layer; *IPL*, inner plexiform layer; *INL*, inner nuclear layer; *OPL*, outer plexiform layer; *ONL*, outer nuclear layer; *PIS*, photoreceptor inner segments; *POS*, photoreceptor outer segments; *BM*, Bruch׳s membrane; *CH*, choroid.

Single transgenic IRBP:HEL mice expressing hen egg lysozyme (HEL) under the retinal interphotoreceptor retinoid-binding protein (IRBP, RBP3) promoter, were generated as previously reported [2]. With age, these mice gradually lose the expression of interphotoreceptor retinoid-binding protein (IRBP; RBP3). In our experiments, we compared 60 day old (“adult”) or 240–300 day old (“aged”) wild type mice (WT; P60 and P240–300, respectively), with said transgenic animals of the same age groups for the cardinal features resembling human atrophic (dry) AMD. Central retinal evaluation by TEM revealed absence of age-related changes occurring in retinas of control adult (P60) WT mice (Fig. 1A-C), while in sTg-IRBP:HEL mice of the same age (Fig. 1D-F) drusen-like deposits were found that accumulated with age (Fig. 1F). These adult mice showed signs of slightly disorganized/shortened photoreceptor layers (Fig. 1D, E) and Bruch׳s membrane thickening with extensive alteration in RPE basal infoldings (Fig. 1F, circle). In terms of retinal degenerative markers, aged control mice (P240–300, WT; Fig. 1G-I) were comparable to adult (P60) sTg-IRBP:Hel mice. In aged sTg-IRBP:HEL mice (P240–300) retinal degeneration became increasingly evident, with complete loss of photoreceptors (Fig. 1J, K) and large sub-RPE drusen-like deposits detected (Fig. 1L). These degenerative features are in agreement with those encountered in human atrophic AMD, thus we propose the sTg-IRBP:HEL mouse model as an accessible and useful vehicle for future AMD research. Data are summarised and described in Table 1, statistics are provided in Table 2.

**Table 1:** specifies photoreceptor inner- (PIS) and outer layer (POS), retinal pigment epithelium (RPE), and Bruch׳s Membrane (BM) thickness as presented in Fig. 1. Medians (50th percentile) and ranges (in brackets) of *n* = 3 measurements each are provided. Wild type (WT; controls) and IRBP:HEL single transgenic (sTg) mice of two age groups were used (P60: post-partum day 60, “adult”; P240–300: post-partum day 240–300, “old”).

	**PIS/POS [µm]**	**RPE [µm]**	**BM [nm]**
**WT P60 (“adult”)**	180.6 (20.8)	5.2 (0.3)	376.3 (172.0)
**WT P240–300 (“old”)**	118.1 (7.0)	4.3 (1.7)	537.6 (215.1)
**sTg-IRBP:HEL P60 (“adult”)**	66.0 (6.9)	3.9 (1.0)	1075.0 (268.8)
**sTg-IRBP:HEL P240–300 (“old”)**	10.4 (3.5)	2.6 (0.7)	806.5 (451.7)

**Table 2:** compares mice groups based on age (P60 vs. P240-300: post-partum day 60, "adult" vs. day 240-300, "old"), or genotype (WT: wildtype vs. sTg-IRBP:HEL: single transgenic). *P*-values are provided; asterisks denote significant differences based on a ≥ 95% level of confidence. Medians (50^th^ percentile) of *n* = 3 independent measurements per marker of interest were compared using the Mann-Whitney *U*-test.

	**PIS/POS, *p*-Value**	**RPE, *p*-Value**	**BM, *p*-Value**
**WT P60 (“adult”)**	0.046*	0.043*	0.127
**WT P240–300 (“old”)**			
**sTg-IRBP:HEL P60 (“adult”)**	0.046*	0.050*	0.050*
**sTg-IRBP:HEL P240–300 (“old”)**			
**WT P60 (“adult”)**	0.050*	0.046*	0.050*
**sTg-IRBP:HEL P60 (“adult”)**			
**WT P240–300 (“old”)**	0.043*	0.178	0.513
**sTg-IRBP:HEL P240–300 (“old”)**			

## 2 Experimental design, materials, and methods

### 2.1 Animals

The generation of sTg-IRBP:HEL mice was previously described [2]. All procedures performed were in agreement with the regulations of the Animal License Act (UK) and followed the ARRIVE guidelines for animal husbandry. All mice were bred in established breeding colonies and maintained/housed in the Medical Research Facility of the University of Aberdeen. Mice genotypes were verified by a routinely used in-house PCR protocol.

### 2.2 Sample preparation for histology, and H&E staining

Wild type mice (B10.BR) and sTg-IRBP:HEL mice of different ages (P60 and P240–300, respectively) were humanely killed and eyes removed immediately. Per age- and test group 2 to 4 animals were analysed. Eyes were fixed in 2.5% *(w/v)* glutaraldehyde (Fisher Chemicals, Loughborough, UK) and embedded in resin to be sectioned for standard H&E staining. Images were recorded using a ProgRes XT Core 5 colour digital microscope camera (JENOPTIK Optical Systems GmbH, Jena, Germany) with samples mounted on an inverted microscope (Axioskop40, Carl Zeiss, MicroImaging GmbH, Jena, Germany).

### 2.3 Sample preparation for TEM

As above, mouse eyes (WT and sTg-IRBP:HEL; P60 and P240–300, respectively) were collected and fixed in 2.5% glutaraldehyde. Anterior segments (*i.e.* cornea, sclera, iris and lens) were removed. The remaining eyecup (vitreous) was then post-fixed in osmium tetroxide, dehydrated in ethanol, followed by acetone, and embedded in Spurr׳s resin. Ultrathin sections were cut using a Leica UC6 microtome (Leica Microsystems), stained with uranyl acetate and lead citrate, and examined with a TEM microscope (JEOL 1400 plus, equipped with an AMT UltraVUE camera).

### 2.4 Statistical analysis

Statistics were performed using IBM SPSS Statistics 25.0. Based on the nature of the data available, the non-parametric procedure of Mann-Whitney U-test was used to compare data based on ranks. Medians (50^th^ percentile) and ranges are presented in Table 1, along with *p*-values based on a ≥ 95% level of confidence in Table 2.
